# Anæsthesia by Ethyl Bromide

**Published:** 1880-09

**Authors:** H. Augustus Wilson

**Affiliations:** Opthalmic and Aural Surgeon to St. Mary's Hospital, and Surgeon in charge of the Surgical Out-patient Department; Lecturer on Microscopic Anatomy and on Fracture Dressings at the Philadelphia School of Anatomy.


THE
AMERICAN JOURNAL
OF
DENTAL SCIENCE.

Vol. XIV. THIRD SERIES—SEPTEMBER, 1880. No. 5.

### ARTICLE I.

### *Anæsthesia by Ethyl Bromide.*

BY H. AUGUSTUS WILSON, M. D.,

Opthalmic and Aural Surgeon to St. Mary's Hospital, and Surgeon in charge of the Surgical Out-patient Department; Lecturer on Microscopic Anatomy and on Fracture Dressings at the Philadelphia School of Anatomy.

I desire to present in this paper a summary of facts known, up to this date, in regard to the anæsthetic use of the ethyl bromide.

During last winter I received a dissection wound, which required laying open; my confidence in the ethyl bromide was such that without the slighest hesitancy I consented to pass under its influence.

Dr. Levis poured one drachm upon a folded napkin, and I with my right hand applied it closely to my face. In one minute Dr. Levis asked if I was ready. I could barely understand what was said, but could not reply, and fearing that the knife would be applied, I managed to utter a groan. A second drachm was applied, and in half a minute more the incision was made, without cognizance on my part, and the napkin removed. One half minute more, or two minutes from the time of commencing to take the anæsthesia I sat up, recognized and called by name a person who had entered the office during my unconscious moments. No nausea or other distressing symptoms arose. My efforts at first were directed to watching the influence it would have upon me, but that influence was exerted so rapidly and stealthily, that ere I was aware of it I was in that happy condition which is such a boon to those about to undergo surgical procedures. Thus my apology is made, if apology is necessary, for presenting this subject again to the profession; my object being to incorporate, if possible, the views expressed in some of the recent articles upon the ethyl bromide and its uses.

The ethyl bromide, which was discovered by Serullas, in 1827, received but little attention until Nunneley, of Leeds, England, called the attention of the profession to it, in 1849. It was not long, however, before he was compelled to abandon it, because of its costliness. Rabutau, of Paris, again evoked interest in the subject, in 1876, in connection wite some experiment on animals.

In 1878 Dr. Laurence Turnbull, of Philadelphia, read a paper before the Pennsylvania State Medical Society, urging the advantages of this article as an anæsthesia.

The matter was looked upon passively until Dr. R. J. Levis, of Philadelphia, took hold of the subject actively, in April 1870, and gave it a trial during his term of service at the Pennsylvania Hospital, and later at the Jefferson College Hospital.

In January, 1880, he published his first article upon the subject. It was not known until after the publication of this article that any experiments on man had previously been made abroad with this agent, and the cautious manner in which each administration was watched showed a desire to understand the *modus operandi* of its application.

That the ethyl bromide occupies its present high standpoint credit is due, first, to Dr. Turnbull, for reviving the attention of the profession to it; and secondly, to Dr. Lewis, who, with his large hospital opportunities and extended experience with anæsthetics, gave the drug a most careful investigation.

Thus, starting from Philadelphia, its application spread to all parts of the country, and recently word has come to us, through the journals, that it has been successfully introduced into Canada and France.

During the past few months a medical journal could hardly be found which did not allude to ethyl bromide, showing conclusively that the profession, tired of the dangerous chloroform, inconvenienced with the slow, nauseating ether, was ready and anxious to accept an article which promised as much as this new candidate for popular favor. Having watched the growth of the use of this anæsthetic from the time it was first used in the Pennsylvania Hospital, frequently administering it for Dr. Levis, in private surgical practice, more frequently, perhaps, carefully noting the effects when administered by Dr. J. B. Roberts, I have continued to use it in preference to any other anæsthetic, at St. Mary's Hospital in this city, and in my private practice.

ITS CHEMISTRY.

Heretofore ethyl bromide has been made by using potassic bromide, but as this was made from ferrious bromide, Dr. Lawrence Wolf, a well-known pharmacist of this city, adapted the following method:—

In a stone jug containing about one gallon of water and two-and-a-half pounds of iron turnings, or wire, five pounds of bromide are gradually added, care being taken not to allow the temperature to rise to high, the jug, besides, being placed in cold or iced water; as soon as the reaction has ceased the solution of the green ferrous bromide is filtered off, the remaining iron being well washed out with warm or boiling water, and to it, in a leaden or glass flask, fifteen pounds of commercial sulphuric acid are added. After the mixture has sufficiently cooled six pints of alcohol (95 per cent.) are intermixed, the mixture well agitated and distilled at a temperature of 200°F., and there maintained until the reaction, which will go on quite lively for a while, shall have ceased, and the ethyl, which has rapidly been gathering in a receiver containing about one ounce of water, has ceased to come over, as can easily be detected when it fails to further sink to the under surface of the layer of water. The ethyl bromide so obtained should be shaken with a solution of potassic bicarbonate, subsequently washed with water and purified by re-distillation.

Dr. Wolff says that he has obtained in this way and from these amounts seven pounds of ethyl bromide, at a cost of not over $4.30, or about 60 cents per pound.

Thus, if ethyl bromide can be manufactured at this very low cost the objection that Nunneley found will not prevent the article from being used.

At the Pennsylvania Hospital the first twenty-five administrations consumed nearly twelve fluid ounces, or about eighteen ounces avoirdupois. The retail price at that time was $3.00 per pound, thus making the total cost $3.37½. The average amount used in each case was 3 9-10 fluid drachms, costing 13½ cents for each patient.

The cost is governed by the manner of using. If given in the bungling and wasteful manner so often seen in the use of other anæsthetic agents, I can readily understand how the cost would be a decided impediment to further use; but if administered as I shall detail later, any one can gain the results obtained above in similar cases.

### ITS PHYSIOLOGY.

There exists such diametrically opposed opinions with reference to the physiology of this agent, that, instead of discussing them, I shall merely allude to the views held by different investigators and await the results of further experiments.

Dr. H. C. Wood, of Philadelphia, deduces from his experiments on animals that anæsthesia may be obtained without reducing the blood pressure, but when given in excess it becomes a distinct depressant to the circulation, reducing the current of blood to a marked extent.

M. Terrillon, of Paris, says that in no case has he observed any phenomena the nature of which would cause fears of asphyxia; neither does ethyl bromide seem to cause syncope.

Dr. Levis observes that as anæthesia progresses the general circulation shows evidences of moderate excitement, indicated by some increase in the rapidity of the action of the heart and increased arterial tension, the face becoming brightly flushed. Nor does he seem inclined to think there is any likelihood of cerebral anæmia or cardiac syncope; and he further observes, that all anæsthetics become eventually, by continuance, depressing agents.

Dr. W. H. Hingston, of Montreal, notices in man an increased frequency in the heart action, together with an increased respiratory movement, and that the pulse and breathing are less influenced than with ether or chloroform.

Dr. Lawrence Wolff, assisted by Dr. J. G. Lee, Physician to the Coroner of Philadelphia, experimented quite largely with rabbits, and observed that those animals which died, to whom respectively ethyl bromide and ether had been administered, presented similar phenomena.

The mode of death appears to have been by gradual paralysis of the cardiac inhibitory motor centres; while the sudden heart failure in the animal to whom was given chloroform, which is typical of chloroform accidents, seems to indicate paralysis of the cardiac motor centres.

Dr. Wolff believes that these experiments go far to show that a direct toxic inueflnce from pure bromide of ethyl need not be apprehended.

Dr. Ott, of Easton, Pa., who has made thorough and scientific researches with the bromide of ethyl, experimenting upon frogs and rabbits, believes that the increased frequency of the pulse is due to stimulation of the accelerative nerves, or of the cardio-motor ganglia, and the dangers in administering the drug are less then those of nitrous oxide.

MODE OF ADMINISTRATION.

It is of great importance that a preliminary drill should be gone through before commencing the administration of any anæthetic, that the patient may know what is required of him and co-operate with the administrator. The assurance of freedom from suffering and danger go far to tranquilize the patient's mind and render him willing to observe such directions as it may be advisable to give him.

I cannot too greatly deprecate the careless manner in which anæsthetics are administered, it being an extremely rare occurrence for the patient to have his heart or lungs examined prior to the administration. A careful physical examination should be instituted in every case about to pass into the anæsthetic state, and should signs be present of affection of either of these organs, the administrator would feel increased responsibility, and would be more watchful for the slightest indication of trouble. But the frequent administration of anæsthetics at the present day, together with the rare occurence of death, has tended to a disregard of danger.

The use of cumbersome mechanical apparatus for the administration of anæsthetics has no advantage over the simple napkin, as used by Dr. Levis.

It is recommended that a folded napkin large enough to cover the entire face be used, and upon the centre of this be pinned several folds of soft linen, about four inches square, upon which is poured, in measured quantities, the anæsthetic.

The patient being upon his back, with his head slightly raised, the preliminary drill having tranquilized him, the napkin is placed over the patient's nose and mouth, and a rapid, decided impression aimed at. The object is to obtain the anæsthetic state as soon as possible, without the intervention of muscular or mental excitement.

The anæsthetic state is usually indicated by a return to normal breathing, resembling that of ordinary sleep.

When this condition is reached the application should be continued only as needed to keep the patient in this condition. It must be remembered that all anæsthetics become depressant when pushed to their toxic effect.

Vomiting is less frequent than in the administration of ether or chloroform—Terrillon, Fere, Hingston, Conner, Roberts, Turnbull, Levis—while Agnew, Haynes, Morton and Prince think that it occurs more frequently.

It seems impossible to reconcile these views, unless allowance is made for the impurity of the earlier samples, and the manner in which administration was conducted; for when given as Dr. Levis recommends, I am certain that vomiting is very much less frequent than when ether is given.

The condition of the patient's stomach is, of course, a very important matter, and it is well to have the patient abstain from solid food for at least four hours, and liquids three hours, before being anæsthetized—(Levis.)

Fere recommends that a fast should be taken from the night before the operation. This may account, in part, at least, for his uniform good results in the administration.

The occurrence or threatening of vomiting during the progress of anæsthesia indicates that the anæsthetic state has not been reached, and an effort should be made to push the ethyl bromide. It must be borne in mind that a fully anæsthetized patient never vomits.

After the completion of the operation, should nausea present itself, small pieces of ice may be ingested, with decided benefit. The patient should be kept in the recumbent position until all tendency to nausea has subsided.

The ethyl bromide is not available, in my experience, in operations about the nose and mouth, when it becomes necessary to suspend the administration for a time, for the rapid elimination alows the patient to return to consciousness, which is apt to be accompanied with more or less struggling.

After complete anæsthesia has been produced by ethyl bromide, I have found it practicable to continue the anæsthesia by the use of sulphuric ether, which has the advantage, and at times the disadvantage, of keeping up the anæsthetic state a considerable time after the napkin has been removed.

In operations lasting over one hour, it is probably safer to keep up the protracted impression with sulphuric ether; but I can conceive of but few operations which would require such a length of time in performing.

The quantity to be used in operation must of necessity be governed by the condition of the patient and skill in the administraton. In a recent case of scirrhus of the mammary gland, upon which Dr. Levis performed excision, I administered seven and one-half drachms of Wyeth's ethyl bromide, keeping the patient completely under its influence for fourty-two minutes. Five minutes after removing the napkin the patient conversed rationally with those about her. In this case there was no nausea, vomiting, or other unpleasant symptom.

The largest amount I have personally known to have been given was eleven drachms during forty minutes, in a case of amputation of the arm, by Dr. Lewis. I am unable to understand the necessity of giving the large quantities said to be required (?) in many of the cases where deleterious defects have been noticed or where anæsthesia could not be obtained.

In the case of Dr. Sims, of New York, it is stated that two ounces had been administered during the first twenty minutes, at the end of which time she vomited freely (showing she was not thoroughly anæsthetized.) The operation (Battey's) lasted one hour and a half, and about five ounces of the ethyl bromide were used.

It is, of course, a well known observation that those accustomed to alcoholic stimulants are with greater difficulty brought under the influence of anæsthetic agents, but this will not account for all the cases where "it became necessary to resort to ether or chloroform."

In reference to the only case of death occuring while under the influence of ethyl bromide, that occured at the Jefferson Hospital, on May, 26th, 1880, I will briefly state that the patient was in a very debilitated condition, but after being in the hospital week after week, with the intention of improving his condition, and warm weather rapidly approaching, he protested against further delay. A consultation being held, an attempt to remove the vesical calculus was deemed advisable.

On May 26, 1880, after the administration of a stimulant and fifteen grains of quinine, two fluid drachms of ethyl bromide were given by the resident surgeon. Soon after the patient struggled, then a third drachm was given. A fourth drachm was given later, and anæsthesia was accomplished. Dr. Levis made the incision, when a gentleman who has acquired a well-desirved reputation as an administrator of anæsthetics called attention to the imperfect respiration. The napkin being removed, the lips were noticed to be of a pinkish color, cyanosis apparently not existing.

Let it suffice to say, that nitrite of amyl, the battery, and artificial respiration were resorted to, and not until an hour had elapsed were the efforts suspended.

The post mortem examination was held two hours after death, by Dr. J. G. Lee, Coroner's physician. Dr. Lee was peculiarly fitted to make the autopsy in this case, inasmuch as he had experimented quite largely with animals, in connection with Dr. Wolff.

He found the following conditions present :—

Commencing rigor mortis. In left perineal region an incision two inches long penetrating skin and superficial fascia. Tissues of scalp congested, membranes of the brain congested, ventriles containing a small amount of clear serum, brain substance normal; membranes of medulla oblongata congested; substance of medulla anæmic- No odor of ethyl bromide perceptible. On opening body, some slight odor of ethyl bromide was noticeable. The apex of left lung was bound to thoracic walls anteriorly and posteriorly by old (circumscribed) pleuritic adhesions. Upper lobe was partially consolidated, the lung tissue containing a number of cavities, with caseous and purelent deposits.

Upper and lower lobes of right lung bound to thoracic walls anteriorly and posteriorly, by old pleuritic adhesions. Lung tissues consolidated and filled with cavities of various sizes. Trachea and bronchi contained a small amount of pus, otherwise normal.

Right side of heart dilated, auricle and ventricle containing post-mortem clots, concentric hypertrophy of left ventricle, which has contracted, and contained a very small post-mortem clot. Left kidney enlarged and diseased; right kidney enlarged. Liver normal. Intestines normal. Concentric hypertrophy of bladder, which contained at its neck two encysted calculi.

Dr. Morris Longstreth, pathologist to the hospital, made microscopic examinations, and reported as follows:—

Kidneys show inflammation of pelvis, extending to entire organ. The morbid process in the lung was catarrhal pneumonia; no tubercular deposits were to be seen.

The coroner's jury rendered the verdict of death from exhaustion, due to disease of the lungs and kidneys.

I think that the case teaches one very important lesson, that a careful physical examination should be instituted in every case, prior to administration of an anæsthetic. Had this been done in this case, I question very much if the operation would have been attempted.

### SOME VIEWS AS TO THE VALUE OF ETHYL BROMIDE AS AN ANÆSTHETIC.

Dr. A. W. Adams, of Colorado, says, from his small experience in its use, that in view of its patent virtues, *i. e.*, its rapid impression, the promptness of recovery from its influence, its agreebleness, and the non-cumbustible character of its vapor, it is worthy of a most extensive trial and investigation.

Dr. J. C. Reeve, of Dayton, Ohio, remarks: "My personal experience with hydrobromic ether fully sustains the observations of others as to its exceeding promptness of action, and the rapidity with which recovery takes place. I can also say that it is pleasanter to inhale than chloroform, which is not very unpleasant, and infinitely pleasanter than ether."

Dr. Woodbury, of Philadelphia. Editorial. "It is probably safer than chloroform, having much the same advantages over ether that chlorotorm has. But let it be understood that there is no such thing as a perfectly safe anæsthetic."

Dr. H. C. Wood. "If any reader will examine the sudden fall of arterial pressure in Experiment 3, and remember the great expensiveness of the bromide, and the lack of any anæsthetic superiority on its part to chloroform, he will, I think, hesitate about endorsing this new agent."

Dr. Ott draws the following conclusions:—

1. "Bromide of ethyl, by ether inhalation or subcutaneous use, kills by a toxic action on the centre of respiration.

2 "That the decrease of force and frequency of the heart contribute to the paralysis of the respiratory centres.

3. "That injections of ethyl into the jugular toward the heart kills by cardiac arrest, probably due to an action on the cardiac muscle.

4. "Bromide of ethyl in toxic doses depresses momentarily the frequency of the heart, followed by a subsequent permanent rise to normal rate.

5. "Bromide of ethyl in toxic doses depresses the actual tension steadily, due in major part to the depressant action of the drug upon the heart; and in minor part to a partial loss of tone of either the spinal vaso-motor centres, or the peripheral vaso-motor system.

6. "The inhibitory power of the pneumogastric is not paralyzed."

Dr. Wolff. "That pure ethyl bromide is, *per se*, an absolutely safe anæsthetic, can, as yet, not be positively stated, but that its action appears to be quite as safe as ether, and certainly more so than the treacherous and dangerous chloroform, seems to us, as a deduction from above related experiments, out of question."

W. H. Hingston, Montreal, Canada, has used no other anæsthetic since commencing the use of bromide of ethyl. There is less resistance and struggling on the part of the patient. Vomiting is less frequent. Eliminated from body more rapidly than any anæsthetic except laughing gas.

"That bromide of ethyl is one of the, and in some respects the, most valuable anæsthetic hitherto used."

Dr. Sims, of New York. "The inference that I draw from the facts in the history of this case is that the anæsthetic was the cause of death, while the manner of death may have been by uræmic poisoning. The lesson from this is, never to give bromide of ethyl in prolonged operations, and never to give it where there is organic disease of the kidneys. What, then, shall we give?"

Dr. Levis. "It is becoming evident that the dread of unavoidable disasters from chloroform and the inconvenience of ether are tending to prevent their humane administration in many cases where the blessing of anæsthesia is due to the sufferer.

"While feeling inclined to impress caution in regard to the use of so powerful an agent as the bromide of ethyl, I am, from a basis of experience, inclined to recommend its use to the profession, and express my conviction that it is practically the best anæsthetic known to the profession."

USES OF ETHYL BROMIDE OTHER THAN ANÆSTHETIC.

Dr. Roberts, of Philadelphia, records a case of angina pectoris to which he was called that yielded quickly to the anæsthetic use of ethyl bromide.

The ethyl bromide has gained a place in obstetric practice, owing to its speedy action. In some recent cases where I have used it, it has given the greatest satisfaction. A single drachm administered at every premonition of a labor pain carried the otherwise suffering woman through it without complete loss of sensibility, and yet in a perfectly painless state, the process of labor apparently not being retarded.

In two cases of lumbago and one of sciatica I have administered the ethyl bromide hypodermically, in doses of ten and fifteen minims, gaining the same results that are obtained by similar applications of chloroform. The patients complained only of a sensation, first of warmth, later of numbness, for some considerable distance about the seat of puncture, which passed off in the course of a few hours. The pain in each case was very speedily relieved, and in the case of sciatica did not return after the third injection, (one having been given on each day).

Dr. Wolff, while testing the action of ethyl bromide upon himself, when taken internally, was suffering with a sick headache, which the drug in question seemed speedily to relieve, and he infers therefrom that cases of nervous irritation and hysteria may receive great benefit from its use.

Dr. Turnbull has used, at his ear clinics, the ethyl bromide, by means of the Politzer bag, for inflating the middle ear, and is much pleased with its action. Upon his recomendation I have used it several times, and am inclined to regard it as a valuable acquisition in the treatment of ear diseases. I have not used it often enough to base any conclusions upon its benefits.

Thus, it will be seen that the ethyl bromide has been used with success as a general anæsthetic in surgery, gyæcology, dentistry, in a case of angina pectoris, and other painful afiections; as a local anæsthetic in nunerous cases not recorded; by inflation in the treatment of tinnitus aurium; internally, for nervous headache; and hypodermically in lumbago and sciatica.

If a medical agent possesses such a large range of usefulness, and when administered judiciously has yielded such good results, while in its infancy, is it not proper that it should be given the benefit of a longer and more cautious trial, that its proper position may be assigned to it?

That it has been useful connot be doubted. That it is capable of producing death should be no bar to its proper use; for, in such view, it would become necessary to strike from our materia medica a long list of remedies without which we would be at a sad loss.—*Medical and Surgical Reporter.*

